# One size does not fit all: towards optimising the therapeutic potential of endogenous pain modulatory systems

**DOI:** 10.1097/j.pain.0000000000002697

**Published:** 2022-05-20

**Authors:** Kirsty Bannister, Sam Hughes

**Affiliations:** a Central Modulation of Pain Lab, Institute of Psychiatry, Psychology and Neuroscience, King's College London, London, United Kingdom; b Pain Modulation Lab, Brain Research, and Imaging Centre (BRIC), School of Psychology, University of Plymouth, Plymouth, United Kingdom

## 1. Introduction

Top–down processing pathways modulate neuronal transmission at the level of the spinal cord dorsal horn and thus govern, in part, the percept of pain. In health, these descending control systems can give rise to a naturally occurring form of pain inhibition. Despite a high therapeutic potential, the clinical applicability of harnessing such endogenous systems has not been fully realised. We propose that this is due to several complicating issues. First, the descending pain modulatory system (DPMS) is influenced by sensory as well as affective brain circuits. Thus, because of the bidirectional nature of the DPMS, pain may be facilitated or inhibited in a manner that corresponds not only to the degree of injury but also to the emotional status of the individual. This relates to the second issue; the DPMS, traditionally viewed as an encompassed “whole” for top–down processing, is in fact comprised of distinct neuronal networks. Emerging data highlight that a blanket pharmacological approach does not consider that diverse circuits, despite overlapping functionality and neurochemistry, require unique intervention. Meaning that, for DPMS-targeted analgesia, activity must be altered in the *relevant* pathway where cortical influences must also be considered.

Here, we review what is known about the neuroanatomical framework by which distinct descending pathways are subserved, citing evidence from both preclinical (animal) and clinical (human) studies. This is timely because movement towards selective therapeutic intervention (ie, relevant to specific features of pathological pain conditions and their comorbidities) will depend on recognition of top–down controls within the DPMS as functionally unique systems that are engaged by specific environmental or affective “drivers.” For this to occur, studies that define the anatomical and pharmacological nature of the circuits therein, and translational delineation of the mechanisms underlying key descending control pathways, are key.

## 2. The descending pain modulatory system

Dissecting the functionality of discrete descending modulatory pathways, where each mechanism likely represents a distinct system, would allow identification of precise and disease-specific novel targets. This has implications for patient stratification (eg, based on correlation or relationships between cognitive or affective processes and pain modulatory pathway efficiency) and related mechanistic therapeutic approaches. Toward this notion, we begin by evidencing the known relationship between “drivers” and neurophysiological systems that affect activity in the DPMS.

### 2.1. Modulators, integrators, and effectors

Endogenous analgesic circuitry can be modulated by pharmacological^3,8,25,30,35,40,72^ or psychological interventions.^10,16,19,36,42,49^ Emotional changes,^23^ mindfulness therapy,^73^ exercise,^22^ and cognitive status^43,68^ may affect brain–spinal cord nociceptive transmission^4^ where convoluted underlying mechanisms influence the centrifugal descending modulation of spinal nociceptive transmission. Take noradrenaline as an example. Opposing alpha 2 adrenoreceptor-mediated facilitatory signalling in the brainstem has been demonstrated,^70^ and the locus coeruleus (LC), a traditionally viewed inhibitory component of the DPMS,^18^ evokes a bidirectional change in thermal nociception in rats when optogenetically activated.^33^ Its modular functional organisation lends the LC to multiple pain-modulatory influences.^9,34,50^ Recently, its propain mechanism has been linked to a regulatory role on noncoerulean noradrenergic cell group(s),^45^ highlighting the functional nuance of seemingly comparable brainstem with spinal cord projection circuits.

Serotonergic, opioidergic, and GABAergic mechanisms are also recruited by descending pathways, in which distinct (even if overlapping) governance of specific circuits occurs. For example, the caudal raphe sends descending projections to the dorsal horn,^37^ and the rostral ventromedial medulla (RVM) harbours neuronal populations involved in antinociceptive and pronociceptive behavioural responses. Most of the transmission is dual GABA and opioidergic.^21^ Interestingly, this unique circuit integrates stress information to limit the presynaptic inhibition of afferent neurons terminating in the same region, representing a network whereby endogenous analgesic processes are influenced by environmental factors. Mechanistically, this links with the influence of higher brain centres on final modulatory output. For example, the forebrain targets caudal medullary regions, and emotional and cognitive processing is implicated in the control of pain transmission through a direct impact on brainstem loci.^51,74^ The dorsal reticular nucleus (DRt) can enhance nociceptive responses,^52^ and circuitry between brainstem sites including the DRt, RVM, and midbrain periaqueductal grey (PAG) provides a substrate for emotional and cognitive modulation of pain, where the targeting of afferent neurons links the “dynamic pain connectome” to integrated pain responses.^53^ Thus, although defining discrete nucleus–spinal cord projection sites is an important first hurdle, identifying their cortical control is crucial for movement towards selective therapeutic interventions.

The rostral anterior cingulate cortex (ACC) explicitly regulates spinal nociceptive processing whereby separable effects on sensory and affective components of the pain descriptive axes are evident,^25,61^ while a basolateral amygdala–prefrontal cortex–PAG–spinal cord pathway that ordinarily drives feed-forward inhibitions contributes to sensory hypersensitivity in animal models of disease.^38^ Amygdala activity influences nocifensive behaviours by projections that affect activity in the pain neuraxis^55,64^ and lateralised outputs from the amygdala and brainstem modulate spinal nociceptive processing by opioidergic mechanisms differentially in health and disease.^60^ This is also evident when considering ACC–spinal cord, right central nucleus of the amygdala–spinal cord, and RVM–spinal cord circuits.^14^ Identifying affective influences on brainstem–spinal cord pathway functionality is complicated; activity in key nuclei may operate as “brake on” or “brake off” systems for the expression of specific circuits. Nonetheless, since defining the top–down regulation of brainstem origin spinally projecting pathways becomes clinically relevant when the anatomical and pharmacological underpinning of those pathways is known, the process is crucial.

## 3. Discrete circuits

External factors can manipulate descending pathway functionality, and yet, there remains an untapped source of clinically relevant targets (linked to mechanisms involved in descending control expression) that could be exploited for improved analgesia. Functional delineation of endogenous pain modulatory systems would allow the development of mechanism-driven therapeutic approaches (for a variety of chronic pain conditions) to be expedited. This is only beneficial, for optimal analgesic prescription, if (1) the circuitry of the pathways being targeted is known and (2) the descending pathways comprising the DPMS have distinct governance.

### 3.1. Descending pain modulatory system pathway governance

Naturally occurring mechanisms by which external factors can manipulate modulatory control expression are exemplified by the application of paradigms that seek to evoke stress-induced analgesia (SIA), diffuse noxious inhibitory controls (DNICs), and offset analgesia (OA), among others. The separable nature of the paradigms evidences the nuanced nature of top–down control activation within the DPMS.

Mechanistically, psychological stress recruits RVM-mediated modulatory mechanisms.^67^ This, coupled with the fact that RVM-driven circuits integrate stress information to limit the presynaptic inhibition of afferent neurons terminating in the same region hints at the functionality (anatomy and pharmacology) of an “SIA descending control pathway.” SIA, a pain suppression system instigated on exposure to unconditioned or conditioned stressful stimuli,^11^ is governed by an interplay between endocannabinoid and endogenous opioid systems in a manner that is specific to the paradigm applied.^2^ More research is required in order that therapeutic targeting strategies for stress and pain-related disease may be finessed. Meanwhile, the noradrenergic DNIC pathway inhibits the spinal neuronal activity when activated by a conditioning stimulus.^7,48^ Conditioned pain modulation (CPM), the proposed human counterpart of DNIC, is dysfunctional in patients with chronic pain ^27,72^ and its maladaptive expression is associated with increased postoperative pain and persistent pain.^71^ Underlying noradrenergic mechanisms explain the relationship between dysfunctional CPM and the beneficial use of tapentadol (μ-opioid receptor agonist and noradrenaline reuptake inhibitor) and duloxetine (serotonin noradrenaline reuptake inhibitor)^62,72^ in a manner that back translates.^6,7^ However, the fact that noradrenergic brainstem–spinal cord pathways are distinctly governed in a “one mechanism does not fit all” manner,^45^ argues that the functionality of discrete pathways is crucial for understanding sensorimotor modulation in health and disease.

The separable nature of specific DPMS-encompassed pathways can also be considered according to those pharmacological mechanisms *not* involved in key circuits. Opioid-independent mechanisms underlie the phenomenon whereby lowered pain scores accompany a small decrease in temperature during noxious thermal stimulation,^54,59^ a paradigm used to evoke OA. Such as SIA and DNIC, OA is a potent example of a situation in which stimulus manipulation induces an adaptive change in sensory processing. Previously, the reduction in pain experience evoked by application of the OA paradigm was correlated with DPMS activity originating in the PAG and RVM in a human fMRI study.^13^ However, the precise mechanistic underpinning is unknown. Onset hyperalgesia (OH) meanwhile describes a situation whereby a disproportionate increase in pain intensity is experienced at a given stimulus intensity when this intensity is preceded by a rise from a lower temperature.^1^ Despite the paradoxical to OA outcome, we hypothesise that OH is a phenomenon of the same pathway but with some separable governance. Indeed, these latest investigations provide evidence for the separable nature of descending modulatory circuits, even those as closely related (for the paradigm applied) as OA and OH.

The clinical benefit of defining DPMS controls will rely in part on optimally prescribing currently available pharmacotherapies according to the dysfunction in question. While mu-opioid receptor expression in the DPMS underlies opioid antinociception^65^ neither morphine nor naloxone impact CPM expression in humans.^20,32^ Previously, pain facilitative CPM observed in patients with fibromyalgia was mechanistically linked to attenuation of pain inhibitory as well as amplification of pain facilitative processes in the central nervous system.^28^ Meanwhile, tapentadol's analgesic mechanism was shown not to extend to an impact on the magnitude of the OA response in diabetic polyneuropathy patients, indicative of a preferential top–down effect for CPM alone.^62^ Cumulatively, the data indicate distinct yet intertwined descending analgesic mechanisms, which are differentially modulated according to the pain state; it is likely the case that multiple endogenous analgesic systems could be dysfunctional in nociplastic patient groups. For example, since CPM is associated with a distinct pharmacological and functional pathway within the DPMS, where wakefulness affects the overall expression status of the mechanistic DNIC underpinning,^5^ it is highly probable that a chronic pain patient with a normal CPM response may have alterations within a separate analgesic system. Thus, optimal analgesic strategies could encompass pharmacological and nonpharmacological interventions that are specific to 1 or 2 cortical and brainstem circuits. This kind of precision medicine requires further translational research efforts; the advent of bedside tests that allow rapid delineation of functionality in key pain processing pathways will first rely on delineation of the pathways in question.

So far, the influence of SIA or DNIC or OA on DPMS functionality and the nature of the underlying mechanisms that lead to dysregulation of DPMS-encompassed vs the specified pathways in chronic pain (where environmental influences on endogenous analgesic processes are likely) are unknown. If, for example, there is a switch in the dominant brainstem noradrenergic nucleus influencing spinal nociceptive processing in chronic pain (or indeed the functionality of reciprocal connection), then the pharmacotherapeutic target has changed. This has implications for age and disease-associated decrements in endogenous analgesic responses, where there is a need to compare pain measures (including activity in discrete modulatory pathways) across the adult lifespan and disease course.^15,44,47^ Plasticity in the pathways comprising the DPMS is a key factor in the transition from acute to chronic pain. Although this topic is outside the scope of the present review, we emphasise that, following the identification of distinct circuits that comprise the DPMS, understanding the nature of the plasticity therein in chronicity (where each circuit, as in health, is likely to undergo changes that are unique to the pathway itself) is going to be a key factor in the eventual optimisation of therapeutically targeting endogenous pain modulatory systems.

## 4. Psychological and environmental influences

Endogenous analgesic circuits may be influenced by psychological and environmental factors; understanding the biomedical mechanisms requires consideration of psychological social theories, further complicating the process of untangling singular controls. Recent NICE guidelines have suggested that nonpharmacological approaches including cognitive behavioural therapy, mindfulness, exercise, and acupuncture should be encouraged even as blanket treatment options for chronic pain conditions driven by centrally mediated changes in the absence of obvious injury (ie, nociplastic pain). Pain catastrophizing is often linked to a vicious cycle of fear avoidance, pain hypervigilance, disability, depression, and ultimately a worsening of chronic pain.^12,69^ Patients with fibromyalgia with high pain catastrophizing scores also showed stronger activation of the ACC suggesting an increased affective processing of their pain,^26^ and patients with knee osteoarthritis with high levels of pain hypervigilance show facilitated temporal summation of pain^31^. Interestingly, mindfulness therapy has been linked with reduced pain catastrophizing in a heterogeneous group of patients with chronic pain,^66^ and a neuroimaging study in healthy volunteers has demonstrated changes in the ACC during a mindfulness paradigm, which was related to a reduction in pain-intensity ratings.^73^ Similar cortical regions have been implicated in pain relief mediated through physical exercise in patients with fibromyalgia, with more activity reported in the dorsolateral PFC following exercise.^17^ Acupuncture has also been shown to cause increased opioid binding in the ACC, which was associated with clinically relevant reduction in pain in patients with fibromyalgia.^29^ Even the ability to mind wander is affiliated with changes in top–down antinociceptive systems and the PAG.^46^ Activation of CPM-dependent mechanisms are also believed to occur during exposure to immersive virtual reality environments, which can also work to attenuate experimentally induced secondary hyperalgesia in human pain models.^43,58^ Taken together, these findings have helped shape our understanding of how psychological therapies known to improve clinical pain symptoms interact with cortical regions implicated in the DPMS. Linking mechanisms of pain relief with activity in a specific circuit is still lacking. However, from these data, an outline therapeutic strategy is formed, whereby application of specific modulatory control–activating paradigms in carefully stratified patient cohorts may, for example, reveal those top–down influences that act as a gatekeeper to the expression of a specific control. This would inform the tools required for back translational studies, where, ideally, patient observations are recapitulated with faithful paradigms at the bench.

## 5. Harnessing cortical influences over descending pain modulatory system pathways

There is a growing body of evidence that suggests harnessing top–down cortical influences over brainstem endogenous analgesic systems could provide a feasible route to precision manipulation of descending control circuits and, thus, precision medicine. Through effective screening for dysfunction in discrete cortically driven cognitive or affective pathways, psychological interventions which target specific DPMS pathways can be tailored to the correct patient. The future of neurotechnology for the treatment of chronic pain also holds promise. Despite the overall poor clinical evidence for transcranial direct current stimulation (tDCS) as a treatment for chronic pain,^63^ it is increasingly apparent that analgesic effect sizes are bigger during dynamic sensory testing paradigms or when sensitisation is present, having minimal effects over acute pain thresholds.^24^ A number of experimental “healthy human” studies have shown that tDCS applied over the primary motor cortex can exert an influence on brainstem regions involved in endogenous pain control,^57^ directly boost CPM efficiency,^19^ and attenuate facilitated spinal pain mechanisms.^39,41,42^ Indeed, baseline CPM efficiency was related to the analgesic effects of tDCS applied over the DLPFC in patients with chronic low back pain,^56^ which others have linked to changes in activity within the posterior insula cortex.^49^ A systems-based approach, whereby screening for specific endogenous analgesic mechanisms (eg, CPM dysfunction), may lead to improved efficacy in future randomised controlled trials. Clinically speaking, optimising the therapeutic potential of these endogenous inhibitory systems through improved patient profiling, and centrally acting targeted interventions, is within reach.

## Conflict of interest statement

The authors have no conflict of interest to declare.

## References

[1] Alter BJ Aung MS Strigo IA Fields HL. Onset hyperalgesia and offset analgesia: transient increases or decreases of noxious thermal stimulus intensity robustly modulate subsequent perceived pain intensity. PLoS One 2020;15:e0231124. 33290407 10.1371/journal.pone.0231124 PMC7723268

[2] Atwal N Winters BL Vaughan CW. Endogenous cannabinoid modulation of restraint stress-induced analgesia in thermal nociception. J Neurochem 2020;152:92–102. 31571215 10.1111/jnc.14884

[3] Baba Y Kohase H Oono Y Fujii-Abe K Arendt-Nielsen L. Effects of dexmedetomidine on conditioned pain modulation in humans. Eur J Pain 2012;16:1137–47. 22392567 10.1002/j.1532-2149.2012.00129.x

[4] Bannister K. Descending pain modulation: influence and impact. Curr Opin Physiol 2019;11:62–6.

[5] Bannister K Kucharczyk MW Graven-Nielsen T Porreca F. Introducing descending control of nociception: a measure of diffuse noxious inhibitory controls in conscious animals. PAIN 2021;162:1957–9. 33470750 10.1097/j.pain.0000000000002203 PMC8205930

[6] Bannister K Lockwood S Goncalves L Patel R Dickenson AH. An investigation into the inhibitory function of serotonin in diffuse noxious inhibitory controls in the neuropathic rat. Eur J Pain 2017;21:750–60. 27891703 10.1002/ejp.979

[7] Bannister K Patel R Goncalves L Townson L Dickenson AH. Diffuse noxious inhibitory controls and nerve injury: restoring an imbalance between descending monoamine inhibitions and facilitations. PAIN 2015;156:1803–11. 26010460 10.1097/j.pain.0000000000000240

[8] Bannister K Qu C Navratilova E Oyarzo J Xie JY King T Dickenson AH Porreca F. Multiple sites and actions of gabapentin-induced relief of ongoing experimental neuropathic pain. PAIN 2017;158:2386–95. 28832395 10.1097/j.pain.0000000000001040 PMC5681862

[9] Brightwell JJ Taylor BK. Noradrenergic neurons in the locus coeruleus contribute to neuropathic pain. Neuroscience 2009;160:174–85. 19223010 10.1016/j.neuroscience.2009.02.023 PMC2677992

[10] Brooks JC Davies WE Pickering AE. Resolving the brainstem contributions to attentional analgesia. J Neurosci 2017;37:2279–91. 28096471 10.1523/JNEUROSCI.2193-16.2016 PMC5354342

[11] Butler RK Finn DP. Stress-induced analgesia. Prog Neurobiol 2009;88:184–202. 19393288 10.1016/j.pneurobio.2009.04.003

[12] Cook AJ Brawer PA Vowles KE. The fear-avoidance model of chronic pain: validation and age analysis using structural equation modeling. PAIN 2006;121:195–206. 16495008 10.1016/j.pain.2005.11.018

[13] Derbyshire SW Osborn J. Offset analgesia is mediated by activation in the region of the periaqueductal grey and rostral ventromedial medulla. Neuroimage 2009;47:1002–6. 19375510 10.1016/j.neuroimage.2009.04.032

[14] Dickenson AH Navratilova E Patel R Porreca F Bannister K. Supraspinal opioid circuits differentially modulate spinal neuronal responses in neuropathic rats. Anesthesiology 2020;132:881–94. 31977518 10.1097/ALN.0000000000003120

[15] Edwards RR Fillingim RB Ness TJ. Age-related differences in endogenous pain modulation: a comparison of diffuse noxious inhibitory controls in healthy older and younger adults. PAIN 2003;101:155–65. 12507710 10.1016/s0304-3959(02)00324-x

[16] Eippert F Bingel U Schoell ED Yacubian J Klinger R Lorenz J Buchel C. Activation of the opioidergic descending pain control system underlies placebo analgesia. Neuron 2009;63:533–43. 19709634 10.1016/j.neuron.2009.07.014

[17] Ellingson LD Stegner AJ Schwabacher IJ Koltyn KF Cook DB. Exercise strengthens central nervous system modulation of pain in fibromyalgia. Brain Sci 2016;6:8. 26927193 10.3390/brainsci6010008 PMC4810178

[18] Fields HL Heinricher MM Mason P. Neurotransmitters in nociceptive modulatory circuits. Annu Rev Neurosci 1991;14:219–45. 1674413 10.1146/annurev.ne.14.030191.001251

[19] Flood A Waddington G Cathcart S. High-definition transcranial direct current stimulation enhances conditioned pain modulation in healthy volunteers: a randomized trial. J Pain 2016;17:600–5. 26844419 10.1016/j.jpain.2016.01.472

[20] France CR Burns JW Gupta RK Buvanendran A Chont M Schuster E Orlowska D Bruehl S. Expectancy effects on conditioned pain modulation are not influenced by naloxone or morphine. Ann Behav Med 2016;50:497–505. 26809850 10.1007/s12160-016-9775-y PMC4935576

[21] Francois A Low SA Sypek EI Christensen AJ Sotoudeh C Beier KT Ramakrishnan C Ritola KD Sharif-Naeini R Deisseroth K Delp SL Malenka RC Luo L Hantman AW Scherrer G. A brainstem-spinal cord inhibitory circuit for mechanical pain modulation by GABA and enkephalins. Neuron 2017;93:822–39. e826. 28162807 10.1016/j.neuron.2017.01.008 PMC7354674

[22] Geva N Defrin R. Enhanced pain modulation among triathletes: a possible explanation for their exceptional capabilities. PAIN 2013;154:2317–23. 23806655 10.1016/j.pain.2013.06.031

[23] Geva N Defrin R. Opposite effects of stress on pain modulation depend on the magnitude of individual stress response. J Pain 2018;19:360–71. 29241836 10.1016/j.jpain.2017.11.011

[24] Giannoni-Luza S Pacheco-Barrios K Cardenas-Rojas A Mejia-Pando PF Luna-Cuadros MA Barouh JL Gnoatto-Medeiros M Candido-Santos L Barra A Caumo W Fregni F. Noninvasive motor cortex stimulation effects on quantitative sensory testing in healthy and chronic pain subjects: a systematic review and meta-analysis. PAIN 2020;161:1955–75. 32453135 10.1097/j.pain.0000000000001893 PMC7679288

[25] Gomtsian L Bannister K Eyde N Robles D Dickenson AH Porreca F Navratilova E. Morphine effects within the rodent anterior cingulate cortex and rostral ventromedial medulla reveal separable modulation of affective and sensory qualities of acute or chronic pain. PAIN 2018;159:2512–21. 30086115 10.1097/j.pain.0000000000001355 PMC6320264

[26] Gracely RH Geisser ME Giesecke T Grant MA Petzke F Williams DA Clauw DJ. Pain catastrophizing and neural responses to pain among persons with fibromyalgia. Brain 2004;127:835–43. 14960499 10.1093/brain/awh098

[27] Graven-Nielsen T Wodehouse T Langford RM Arendt-Nielsen L Kidd BL. Normalization of widespread hyperesthesia and facilitated spatial summation of deep-tissue pain in knee osteoarthritis patients after knee replacement. Arthritis Rheum 2012;64:2907–16. 22421811 10.1002/art.34466

[28] Harper DE Ichesco E Schrepf A Hampson JP Clauw DJ Schmidt-Wilcke T Harris RE Harte SE. Resting functional connectivity of the periaqueductal gray is associated with normal inhibition and pathological facilitation in conditioned pain modulation. J Pain 2018;19:635 e1–e15. 10.1016/j.jpain.2018.01.001 PMC5972067 29360608

[29] Harris RE Zubieta JK Scott DJ Napadow V Gracely RH Clauw DJ. Traditional Chinese acupuncture and placebo (sham) acupuncture are differentiated by their effects on mu-opioid receptors (MORs). Neuroimage 2009;47:1077–85. 19501658 10.1016/j.neuroimage.2009.05.083 PMC2757074

[30] Hayashida K Obata H Nakajima K Eisenach JC. Gabapentin acts within the locus coeruleus to alleviate neuropathic pain. Anesthesiology 2008;109:1077–84. 19034104 10.1097/ALN.0b013e31818dac9c PMC2843419

[31] Herbert MS Goodin BR Pero STt Schmidt JK Sotolongo A Bulls HW Glover TL King CD Sibille KT Cruz-Almeida Y Staud R Fessler BJ Bradley LA Fillingim RB. Pain hypervigilance is associated with greater clinical pain severity and enhanced experimental pain sensitivity among adults with symptomatic knee osteoarthritis. Ann Behav Med 2014;48:50–60. 24352850 10.1007/s12160-013-9563-x PMC4063898

[32] Hermans L Nijs J Calders P De Clerck L Moorkens G Hans G Grosemans S Roman De Mettelinge T Tuynman J Meeus M. Influence of morphine and naloxone on pain modulation in rheumatoid arthritis, chronic fatigue syndrome/fibromyalgia, and controls: a double-blind, randomized, placebo-controlled, cross-over study. Pain Pract 2018;18:418–30. 28722815 10.1111/papr.12613

[33] Hickey L Li Y Fyson SJ Watson TC Perrins R Hewinson J Teschemacher AG Furue H Lumb BM Pickering AE. Optoactivation of locus ceruleus neurons evokes bidirectional changes in thermal nociception in rats. J Neurosci 2014;34:4148–60. 24647936 10.1523/JNEUROSCI.4835-13.2014 PMC3960461

[34] Hirschberg S Li Y Randall A Kremer EJ Pickering AE. Functional dichotomy in spinal- vs prefrontal-projecting locus coeruleus modules splits descending noradrenergic analgesia from ascending aversion and anxiety in rats. Elife 2017;6:e29808. 29027903 10.7554/eLife.29808 PMC5653237

[35] Hodkinson DJ Khawaja N O'Daly O Thacker MA Zelaya FO Wooldridge CL Renton TF Williams SC Howard MA. Cerebral analgesic response to nonsteroidal anti-inflammatory drug ibuprofen. PAIN 2015;156:1301–10. 25851460 10.1097/j.pain.0000000000000176

[36] Hoffman HG Richards TL Bills AR Van Oostrom T Magula J Seibel EJ Sharar SR. Using FMRI to study the neural correlates of virtual reality analgesia. CNS Spectr 2006;11:45–51. 16400255 10.1017/s1092852900024202

[37] Hornung JP. The human raphe nuclei and the serotonergic system. J Chem Neuroanat 2003;26:331–43. 14729135 10.1016/j.jchemneu.2003.10.002

[38] Huang J Gadotti VM Chen L Souza IA Huang S Wang D Ramakrishnan C Deisseroth K Zhang Z Zamponi GW. A neuronal circuit for activating descending modulation of neuropathic pain. Nat Neurosci 2019;22:1659–68. 31501573 10.1038/s41593-019-0481-5

[39] Hughes S Grimsey S Strutton PH. Primary motor cortex transcranial direct current stimulation modulates temporal summation of the nociceptive withdrawal reflex in healthy subjects. Pain Med 2019;20:1156–65. 30535165 10.1093/pm/pny200

[40] Hughes S Hickey L Donaldson LF Lumb BM Pickering AE. Intrathecal reboxetine suppresses evoked and ongoing neuropathic pain behaviours by restoring spinal noradrenergic inhibitory tone. PAIN 2015;156:328–34. 25599454 10.1097/01.j.pain.0000460313.73358.31

[41] Hughes SW Ali M Sharma P Insan N Strutton PH. Frequency-dependent top-down modulation of temporal summation by anodal transcranial direct-current stimulation of the primary motor cortex in healthy adults. Eur J Pain 2018. doi: 10.1002/ejp.1238 [Epub ahead of print]. 29704875

[42] Hughes SW Ward G Strutton PH. Anodal transcranial direct current stimulation over the primary motor cortex attenuates capsaicin-induced dynamic mechanical allodynia and mechanical pain sensitivity in humans. Eur J Pain 2020;24:1130–7. 32170802 10.1002/ejp.1557

[43] Hughes SW Zhao H Auvinet EJ Strutton PH. Attenuation of capsaicin-induced ongoing pain and secondary hyperalgesia during exposure to an immersive virtual reality environment. Pain Rep 2019;4:e790. 31984295 10.1097/PR9.0000000000000790 PMC6903343

[44] Kucharczyk MW Derrien D Dickenson AH Bannister K. The stage-specific plasticity of descending modulatory controls in a rodent model of cancer-induced bone pain. Cancers (Basel) 2020;12:3286. 33172040 10.3390/cancers12113286 PMC7716240

[45] Kucharczyk MW Di Domenico F Bannister K. Distinct brainstem to spinal cord noradrenergic pathways inversely regulate spinal neuronal activity. Brain 2022. doi: 10.1093/brain/awac085 [Epub ahead of print]. PMC9337805 35245374

[46] Kucyi A Salomons TV Davis KD. Mind wandering away from pain dynamically engages antinociceptive and default mode brain networks. Proc Natl Acad Sci U S A 2013;110:18692–7. 24167282 10.1073/pnas.1312902110 PMC3832014

[47] Lariviere M Goffaux P Marchand S Julien N. Changes in pain perception and descending inhibitory controls start at middle age in healthy adults. Clin J Pain 2007;23:506–10. 17575490 10.1097/AJP.0b013e31806a23e8

[48] Le Bars D Dickenson AH Besson JM. Diffuse noxious inhibitory controls (DNIC). I. Effects on dorsal horn convergent neurones in the rat. PAIN 1979;6:283–304. 460935 10.1016/0304-3959(79)90049-6

[49] Lin RL Douaud G Filippini N Okell TW Stagg CJ Tracey I. Structural connectivity variances underlie functional and behavioral changes during pain relief induced by neuromodulation. Sci Rep 2017;7:41603. 28148969 10.1038/srep41603 PMC5288647

[50] Llorca-Torralba M Borges G Neto F Mico JA Berrocoso E. Noradrenergic Locus Coeruleus pathways in pain modulation. Neuroscience 2016;338:93–113. 27267247 10.1016/j.neuroscience.2016.05.057

[51] Llorca-Torralba M Suarez-Pereira I Bravo L Camarena-Delgado C Garcia-Partida JA Mico JA Berrocoso E. Chemogenetic silencing of the locus coeruleus-basolateral amygdala pathway abolishes pain-induced anxiety and enhanced aversive learning in rats. Biol Psychiatry 2019;85:1021–35. 30987747 10.1016/j.biopsych.2019.02.018

[52] Martins I de Vries MG Teixeira-Pinto A Fadel J Wilson SP Westerink BH Tavares I. Noradrenaline increases pain facilitation from the brain during inflammatory pain. Neuropharmacology 2013;71:299–307. 23602988 10.1016/j.neuropharm.2013.04.007

[53] Martins I Tavares I. Reticular formation and pain: the past and the future. Front Neuroanat 2017;11:51. 28725185 10.3389/fnana.2017.00051 PMC5497058

[54] Martucci KT Eisenach JC Tong C Coghill RC. Opioid-independent mechanisms supporting offset analgesia and temporal sharpening of nociceptive information. PAIN 2012;153:1232–43. 22503222 10.1016/j.pain.2012.02.035 PMC3358522

[55] McGaraughty S Farr DA Heinricher MM. Lesions of the periaqueductal gray disrupt input to the rostral ventromedial medulla following microinjections of morphine into the medial or basolateral nuclei of the amygdala. Brain Res 2004;1009:223–7. 15120601 10.1016/j.brainres.2004.02.048

[56] McPhee ME Graven-Nielsen T. Medial prefrontal high-definition transcranial direct current stimulation to improve pain modulation in chronic low back pain: a pilot randomized double-blinded placebo-controlled crossover trial. J Pain 2021;22:952–67. 33676009 10.1016/j.jpain.2021.02.012

[57] Meeker TJ Keaser ML Khan SA Gullapalli RP Seminowicz DA Greenspan JD. Non-invasive motor cortex neuromodulation reduces secondary hyperalgesia and enhances activation of the descending pain modulatory network. Front Neurosci 2019;13:467. 31139047 10.3389/fnins.2019.00467 PMC6519323

[58] Mehesz E Karoui H Strutton PH Hughes SW. Exposure to an immersive virtual reality environment can modulate perceptual correlates of endogenous analgesia and central sensitization in healthy volunteers. J Pain 2021. doi: 10.1016/j.jpain.2020.12.007 [Epub ahead of print]. 33465506

[59] Naugle KM Cruz-Almeida Y Fillingim RB Riley JL III. Offset analgesia is reduced in older adults. PAIN 2013;154:2381–7. 23872117 10.1016/j.pain.2013.07.015 PMC3894599

[60] Navratilova E Nation K Remeniuk B Neugebauer V Bannister K Dickenson AH Porreca F. Selective modulation of tonic aversive qualities of neuropathic pain by morphine in the central nucleus of the amygdala requires endogenous opioid signaling in the anterior cingulate cortex. PAIN 2020;161:609–18. 31725062 10.1097/j.pain.0000000000001748 PMC7124010

[61] Navratilova E Xie JY Meske D Qu C Morimura K Okun A Arakawa N Ossipov M Fields HL Porreca F. Endogenous opioid activity in the anterior cingulate cortex is required for relief of pain. J Neurosci 2015;35:7264–71. 25948274 10.1523/JNEUROSCI.3862-14.2015 PMC4420787

[62] Niesters M Proto PL Aarts L Sarton EY Drewes AM Dahan A. Tapentadol potentiates descending pain inhibition in chronic pain patients with diabetic polyneuropathy. Br J Anaesth 2014;113:148–56. 24713310 10.1093/bja/aeu056

[63] O'Connell NE Marston L Spencer S DeSouza LH Wand BM. Non-invasive brain stimulation techniques for chronic pain. Cochrane Database Syst Rev 2018;4:CD008208. 29652088 10.1002/14651858.CD008208.pub5 PMC6494527

[64] Phelps CE Navratilova E Dickenson AH Porreca F Bannister K. Kappa opioid signaling in the right central amygdala causes hind paw specific loss of diffuse noxious inhibitory controls in experimental neuropathic pain. PAIN 2019;160:1614–21. 30870321 10.1097/j.pain.0000000000001553

[65] Pinto M Castro AR Tshudy F Wilson SP Lima D Tavares I. Opioids modulate pain facilitation from the dorsal reticular nucleus. Mol Cell Neurosci 2008;39:508–18. 18725300 10.1016/j.mcn.2008.07.008

[66] Schutze R Rees C Preece M Schutze M. Low mindfulness predicts pain catastrophizing in a fear-avoidance model of chronic pain. PAIN 2010;148:120–7. 19944534 10.1016/j.pain.2009.10.030

[67] Terman GW Shavit Y Lewis JW Cannon JT Liebeskind JC. Intrinsic mechanisms of pain inhibition: activation by stress. Science 1984;226:1270–7. 6505691 10.1126/science.6505691

[68] Torta DM De Laurentis M Eichin KN von Leupoldt A van den Broeke EN Vlaeyen JWS. A highly cognitive demanding working memory task may prevent the development of nociceptive hypersensitivity. PAIN 2020;161:1459–69. 32102023 10.1097/j.pain.0000000000001841

[69] Vlaeyen JWS Kole-Snijders AMJ Boeren RGB van Eek H. Fear of movement/(re)injury in chronic low back pain and its relation to behavioral performance. PAIN 1995;62:363–72. 8657437 10.1016/0304-3959(94)00279-N

[70] Wei H Pertovaara A. Spinal and pontine alpha2-adrenoceptors have opposite effects on pain-related behavior in the neuropathic rat. Eur J Pharmacol 2006;551:41–9. 17027962 10.1016/j.ejphar.2006.08.064

[71] Yarnitsky D. Conditioned pain modulation (the diffuse noxious inhibitory control-like effect): its relevance for acute and chronic pain states. Curr Opin Anaesthesiol 2010;23:611–15. 20543676 10.1097/ACO.0b013e32833c348b

[72] Yarnitsky D Granot M Nahman-Averbuch H Khamaisi M Granovsky Y. Conditioned pain modulation predicts duloxetine efficacy in painful diabetic neuropathy. PAIN 2012;153:1193–8. 22480803 10.1016/j.pain.2012.02.021

[73] Zeidan F Martucci KT Kraft RA Gordon NS McHaffie JG Coghill RC. Brain mechanisms supporting the modulation of pain by mindfulness meditation. J Neurosci 2011;31:5540–8. 21471390 10.1523/JNEUROSCI.5791-10.2011 PMC3090218

[74] Zhang L Zhang Y Zhao ZQ. Anterior cingulate cortex contributes to the descending facilitatory modulation of pain via dorsal reticular nucleus. Eur J Neurosci 2005;22:1141–8. 16176356 10.1111/j.1460-9568.2005.04302.x

